# Neuroimaging insights into recent suicide attempters utilizing the raven task

**DOI:** 10.1371/journal.pone.0327562

**Published:** 2025-08-04

**Authors:** Morteza Fattahi, Milad Esmaeil-Zadeh, Hamid Soltanian-Zadeh, Nafee Rasouli, Niloofar Fallahinia, Amirhossein Jafari Mehdi Abad, Reza Khosrowabadi, Seyed Kazem Malakouti

**Affiliations:** 1 Control and Intelligent Processing Center of Excellence (CIPCE), School of Electrical and Computer Engineering, College,; 2 Department of Clinical Psychology, School of Behavioral Sciences and Mental Health, Iran University of Medical Sciences, Tehran, Iran; 3 Mental Health Research Center, Iran University of Medical Sciences, Tehran, Iran; 4 Department of Psychiatry, Roozbeh hospital, School of Medicine, Tehran University of Medical Sciences, Tehran, Iran; 5 Institute for Cognitive and Brain Sciences, Shahid Beheshti University, Tehran, Iran; 6 Geriatric Mental Health Research Center, School of Behavioral Sciences and Mental Health, Iran University of Medical Sciences, Tehran, Iran; Hochschule Niederrhein - Campus Mönchengladbach: Hochschule Niederrhein - Campus Monchengladbach, GERMANY

## Abstract

**Background:**

Understanding brain function in individuals who have recently attempted suicide is critical for improving diagnosis and treatment strategies. This study aimed to examine neural activity patterns in such individuals (who had attempted suicide 1–4 weeks before the study) using functional magnetic resonance imaging (fMRI) and the General Linear Model (GLM).

**Methods:**

Sixty participants were recruited and categorized into three groups: individuals with a recent suicide attempt and Major Depressive Disorder (SA + MDD), individuals with Major Depressive Disorder without a suicide attempt (MDD), and healthy controls (HC). Participants performed the Raven task, consisting of 24 trials with a 25-second time limit for each item. Brain activity was analyzed to identify regional differences among groups using ANOVA, followed by Tukey’s post-hoc pairwise comparisons.

**Results:**

Compared to the MDD and HC groups, the SA + MDD group exhibited significantly reduced activation in the left medial superior frontal cortex (SFC), left anterior cingulate cortex (ACC), and left precentral gyrus. Positive correlations were observed between the Scale for Suicidal Ideation scores and activity in the medial SFC and ACC, whereas a negative correlation was found with precentral gyrus activity. While task accuracy did not significantly differ among the groups, the SA + MDD group demonstrated significantly shorter response durations.

**Conclusion:**

Individuals with recent suicide attempts show diminished activation in key left-hemispheric regions involved in cognitive control and problem-solving, including the medial SFC, ACC, and precentral gyrus. These neural deficits may impair decision-making and problem-solving abilities, particularly when compounded by hopelessness and a diminished sense of purpose, potentially contributing to increased suicide risk.

## Introduction

Depression is strongly associated with elevated suicide rates, with approximately 50% of individuals who die by suicide having been diagnosed with this condition [1]. Beyond its emotional and psychological toll, depression is also linked to a range of physiological consequences and cognitive impairments [2]. Among those affected by depression, maintaining healthy interpersonal relationships can prove to be particularly challenging, often resulting in significant difficulties in social, psychological, and intellectual functioning [3].

Extensive research has demonstrated that psychological functioning is closely related to mood disorders, such as major depressive disorder (MDD), particularly in individuals with MDD who have made suicide attempts. As such, it is essential to investigate the psychological and cognitive processes in these individuals and compare them to healthy controls. One tool often employed to assess cognitive function is the Raven Progressive Matrices (RPM) test, which measures general cognitive ability, commonly referred to as “decision-making” capability. This test evaluates the mental strategies required to solve problems and provides valuable insights into an individual’s cognitive function [4].

Recent studies indicate that depression can significantly impair psychological and intellectual functioning, impacting various aspects of life, including work performance, social interactions, and overall personal role fulfillment. Evidence suggests that the severity of depression negatively correlates with psychological functioning, with greater impairment observed as the intensity of the disorder increases. Furthermore, research on individuals with depression who have attempted suicide reveals a tendency toward cognitive rigidity, where these individuals face difficulties in identifying, generating, and implementing alternative solutions to problems. This cognitive inflexibility suggests a limited capacity for adaptive problem-solving [5].

Given these insights, it is critical to investigate brain activity in individuals with depression while they perform cognitive tasks such as the Raven Progressive Matrices. Comparing the brain activity of depressed individuals—particularly those with a history of suicide attempts—to that of healthy individuals can reveal significant differences in brain region activation, thereby contributing to a deeper understanding of the cognitive and neural mechanisms underlying depression and suicide risk.

Functional Magnetic Resonance Imaging (fMRI) is a sophisticated neuroimaging technique that measures changes in Blood Oxygen Level-Dependent (BOLD) signals, offering valuable insights into the neural activity underlying various mental processes, including psychological disorders. In particular, research into the neurobiology of suicide has identified several brain regions that are implicated in suicidal behaviors. Notably, regions such as the insular cortex and the anterior cingulate cortex (ACC) exhibit both hyperactivity and hypoactivity in association with suicidal tendencies [6].

Numerous resting-state and task-based fMRI studies have been conducted to explore the neural correlates of suicidal behavior. One such study employed resting-state functional connectivity and computational modeling to investigate differences between individuals who had attempted suicide and those who had only experienced high levels of suicidal ideation. The results indicated that dysfunction within specific neural areas of the insula may play a crucial role in the transition from suicidal thoughts to suicidal actions, potentially through increased psychological pain perception and a diminished sensitivity to physical harm [7].

Multidomain functional neuroimaging tasks, such as the Cyberball Game and the Go-No-Go task, have proven effective in identifying past suicidal behaviors beyond clinical assessments. One study found that adolescents with a history of suicidal behaviors exhibited reduced activity in the left insula during social entry tasks compared to both healthy controls and MDD patients. Additionally, these adolescents displayed heightened activity in the right medial prefrontal gyrus during social rejection and increased activity in the bilateral precentral gyrus during response inhibition tasks. These findings suggest that alterations in these brain regions may be associated with the emergence of suicidal behaviors in adolescence [8].

Another study examined cognitive performance in planning and working memory tasks, revealing distinct changes in prefrontal brain activation patterns in patients with Major Depressive Disorder (MDD) compared to healthy controls. These results contribute to a deeper understanding of the neural mechanisms underlying MDD and its impact on cognitive functions [9]. Furthermore, a review of neuroimaging studies highlights the role of the ACC in suicidal behavior, with both hyperactivity and hypoactivity observed depending on task conditions and the nature of stimuli [10]. However, further research is needed to elucidate the precise role of the ACC in relation to suicidal tendencies.

In one study, researchers compared brain functions in MDD patients with a history of suicide attempts, those with suicidal ideation but no suicide attempts, and a control group of MDD patients without any suicidal behaviors. The results revealed that patients with a history of suicide attempts exhibited significantly reduced activity in the bilateral fusiform gyrus during emotional face processing compared to both other groups. However, no notable differences were observed in executive planning tasks across the groups [6].

The present study aims to identify distinct brain regions that exhibit significant differences among three groups—MDD patients with a history of suicide attempts, MDD patients without a history of suicide, and healthy controls—during the Raven task. Additionally, we seek to explore how these differences are related to the participants’ respective conditions and cognitive abilities.

## Method

**Fig 1:**
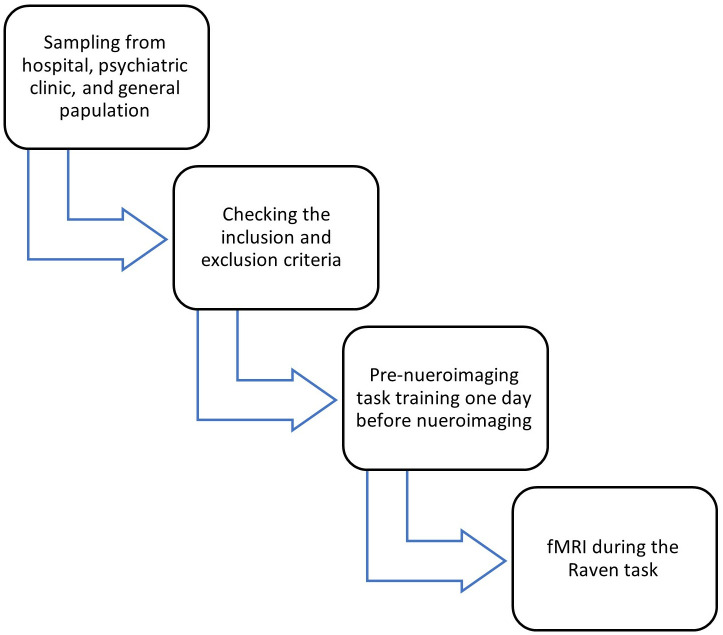
Flow chart of implementation steps.

This study employed a case-control design. Participants who had attempted suicide within the past 1–4 weeks were recruited from the Poisoning Department of Baharloo Hospital, a specialized center for suicide and toxicology cases. Recruitment commenced on June 9, 2021, and continued until October 17, 2022. Eligible patients, referred by hospital psychiatrists, were selected by clinical psychologists according to well-defined inclusion and exclusion criteria (Fig 1).

### Ethics statement

This study was approved by the Institutional Review Board (IRB) of the National Institute for Medical Research Development (NIMAD), under approval ID IR.NIMAD.REC.1398.158. Additionally, approval was granted by Ehsan Shamsi Gooshki, Chair of the Regional Research Ethics Committee.

### Participants

The study initially included 62 participants, divided into three groups: 20 individuals in the Attempted Suicide + Major Depressive Disorder (SA + MDD) group, 21 individuals in the Major Depressive Disorder (MDD) patient control group, and 21 healthy controls. However, 2 participants were excluded during the analysis to ensure equal group sizes for performing Tukey post hoc analysis. Consequently, the final cohort consisted of 20 participants in each group. All participants were between the ages of 18 and 55 and were matched on chronological age, gender, and educational background.

Prior to participation, all individuals provided written informed consent. They also underwent two separate training sessions to familiarize themselves with the tasks to be performed during functional magnetic resonance imaging (fMRI).

Participants in the SA + MDD group were recruited from the Poisoning Department of Baharloo Hospital. These patients, admitted due to suicide attempts, were referred by a psychiatrist. Relevant data, including the time of the suicide attempt and hospitalization, were extracted from their medical records. Neuroimaging was conducted approximately one week after discharge. The inclusion criteria for the SA + MDD group were: 1) a suicide attempt within the past four weeks, 2) a current diagnosis of MDD according to DSM-5, and 3) a Beck Depression Inventory (BDI) score of 23 or higher. Exclusion criteria included: psychotic or bipolar disorders, a history of substance or alcohol abuse, severe head trauma, central nervous system disorders, and a Beck Suicide Intent Scale (BSI) score below 15. The MDD patient control group consisted of individuals referred by psychiatrists who met the criteria for MDD and had a BDI score of 23 or higher but without any personal history of suicidal behavior. Healthy controls were selected from the general population and had no personal or family history of psychiatric disorders or suicide attempts. They were matched for age, gender, and educational background to the SA + MDD and MDD groups, ensuring a well-balanced representation across all groups.

### The Raven Task

**Fig 2:**
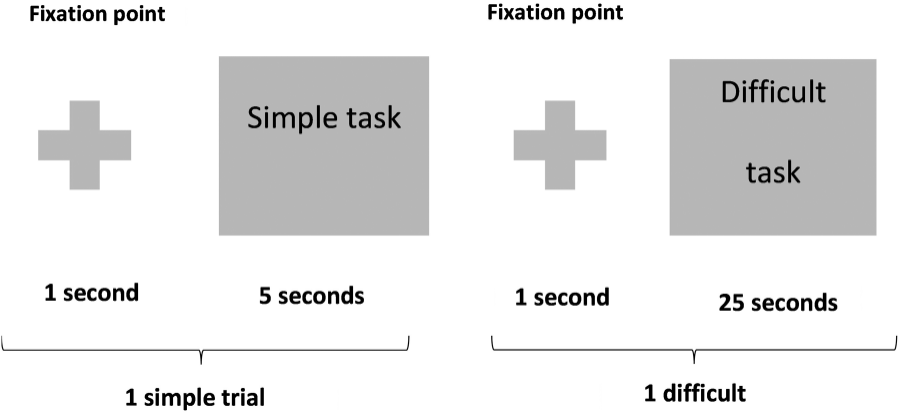
24 trials of the Raven task, including simple and difficult trials.

The Raven’s Progressive Matrices (RPM) were utilized to assess abstract reasoning and fluid intelligence. These matrices consist of visual geometric patterns with a missing piece, and participants are required to select the correct answer from a set of options, based on underlying patterns or rules. Participants completed a total of 24 trials, with each trial offering four possible answers. For each trial, participants were given 25 seconds to complete the Raven-difficult tasks and 5 seconds for the Raven-simple tasks, following the standardized procedures employed in previous studies [11–13]. Two example trials from the Raven task are shown in Fig 2. The difficult and simple tasks were presented in a randomized order.

In this study, only the results from the difficult tasks are analyzed and reported. During both the simple and difficult tasks, participants were instructed to respond by pressing a designated button at their discretion.

### Self-report questionnaires

#### Beck hopelessness scale (BHS).

The Beck Hopelessness Scale is a self-report questionnaire [11], and its Persian version has high reliability (r = 0.70) and internal consistency (Cronbach’s alpha = 0.71) [12].

#### Beck scale for suicidal ideation (BSSI).

The Self-reporting edition of BSSI [13] is a 19-item instrument that assesses the severity of suicidal thoughts. The psychometric properties of the Persian version of BSSI are approved for use in research settings [14].

#### Barratt’s impulsivity scale (BIS-11).

BIS-11 is a self-report questionnaire designed to evaluate impulsiveness. It was developed by Barratt in 1990 [15]. Reliability was assessed using Cronbach’s alpha and test-retest methods, yielding values of 0.81 and 0.77, respectively. The study confirms that BIS-11 is applicable for Iranian samples [16].

#### Buss perry aggression questionnaire (BPAQ).

The Buss-Perry Aggression Questionnaire (BPAQ) has 29 items and measures global aggression [17]. Samani et al. evaluated the Persian version of BPAQ and found it to have appropriate test-retest reliability (0.78) with all items having a significant correlation with the total score except for item 29 (0.25 to 0.52) [18].

#### Reasons for living inventory (RFL).

The RFL is a 48-item inventory that assesses factors that protect against suicide [19]. The Persian version was standardized with a high test-retest coefficient of 0.93 and a Cronbach’s alpha of 0.95 [20].

#### Spielberger anxiety state-trait inventory (STAI).

STAI is a 40-item inventory that evaluates state and trait anxiety [21]. A study on the Persian version of STAI-Y showed high internal consistency with Cronbach’s alpha of 0.88 and 0.84 for trait and state anxiety, respectively. The convergent validity of BAI and STAI-Y for both state and trait anxiety was found to be strong [22].

#### Beck depression inventory (BDI-II).

BDI-II is a tool used to assess severity of depression [23]. A study evaluated the Persian version of BDI-II and found high consistency (alpha = 0.87) and reliable results (r = 0.74) with a strong correlation to the Automatic Thoughts Questionnaire (ATQ). A cut-off point of 23 was used to screen depression severity [24].

### Neuroimaging

This study employed a simultaneous electroencephalography (EEG) and functional magnetic resonance imaging (fMRI) design. The results of the EEG component have been published separately [25]. MRI data were acquired using a Siemens 3.0 Tesla scanner at the Iranian National Brain Mapping Lab (NBML) with a 64-channel head coil. T1-weighted structural images were obtained using an MPRAGE protocol. The imaging parameters for these scans included a repetition time (TR) of 1810 ms, an echo time (TE) of 3.47 ms, a slice thickness of 1.0 mm, 176 total slices, a phase encoding direction of ROW, and a matrix size of 256 × 256 × 176. For the fMRI data, an echo-planar imaging (EPI) sequence was utilized with the following parameters: repetition time (TR) of 3000 ms, flip angle of 80°, 220 time points, echo time (TE) of 30 ms, and 44 axial slices.

### Behavioral data

The behavioral analysis focused on evaluating participants’ performance on the Raven task. Specifically, we measured the number of correct responses and the response time, recorded in milliseconds. In addition, the total number of correct responses for each participant was calculated to assess their reasoning abilities. To ensure data accuracy, outliers in both reaction time (RT) and number of correct responses (NCR) were identified using a three-standard deviation (SD) criterion. Importantly, no outliers were detected in either dataset. The average reaction time and average number of correct responses were then calculated for each participant, and subsequent analyses were conducted based on these metrics.

### fMRI data processing

All preprocessing steps were performed using SPM12 within the MATLAB environment, as outlined below:

**Slice-Time Correction**: Temporal differences between slices within a single volume were corrected to ensure consistent timing across the brain.

**Realignment**: Head motion during scanning was corrected by realigning the slice-time corrected data. Participants exhibiting excessive motion (greater than 3 degrees rotationally or greater than half a voxel size in any direction) were excluded from the study. Each image in the time series was aligned to a reference image, chosen as the first image of the series.

**Coregistration**: Functional and anatomical data were aligned to ensure accurate correspondence between fMRI images and structural images, ensuring that the functional data accurately represented the anatomical structures.

**Normalization**: The individual brain images were transformed into a standard space (MNI space in this study) to facilitate group analysis. This step involved spatially warping the images to match a common anatomical template, allowing for comparisons across participants.

**Smoothing**: Spatial smoothing was applied using a Gaussian kernel (full width at half maximum = 6 mm) to increase the signal-to-noise ratio and account for individual anatomical variations, thereby enhancing statistical power and promoting a more normal distribution of the data.

**High-Pass Filtering**: A high-pass filter with a cutoff of 128 seconds was applied to mitigate low-frequency drifts in the data.

To analyze the preprocessed fMRI data, we used a standard general linear model (GLM) approach implemented in SPM12 (Wellcome Trust Centre for Neuroimaging, London, UK). A design matrix was constructed to specify the timing and duration of each experimental condition. The GLM was then fitted to the preprocessed data, yielding beta values representing the brain’s response to each condition. Contrasts were defined to test specific hypotheses, such as comparing brain activity during task conditions versus rest conditions. Statistical inference was performed on the resulting statistical maps, identifying brain regions showing significant changes in activity corresponding to the defined contrasts.

To address the multiple comparisons problem, we initially planned to apply a false discovery rate (FDR) correction. However, due to the lack of significant findings after preprocessing, we opted to focus on meaningful activation patterns without applying an FDR correction. Instead, we applied a threshold based on practical significance, allowing us to examine potential activations without being overly influenced by multiple comparison corrections. All analyses were conducted within the MATLAB 2021b environment.

**Fig 3:**
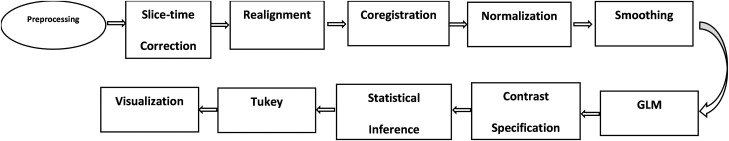
A schematic overview of the fMRI data processing pipeline employed in this study.

After performing the multivariate test, Tukey post-hoc analysis was conducted to assess specific group differences. This analysis was carried out using DPABI version 5.1. A schematic overview of the fMRI data processing pipeline is provided in Fig 3.

### Statistical analysis

Demographic and clinical characteristics were summarized using descriptive statistics. A one-way analysis of variance (ANOVA) was performed with SPSS version 24 to compare the means of clinical variables, followed by Tukey post-hoc pairwise comparisons. To identify significantly different brain regions among the three groups, an ANOVA was conducted using DPABI version 5.1, with a significance threshold set at a p-value of 0.01.

It is important to note that after applying false discovery rate (FDR) and family-wise error (FWE) corrections at a p-value of 0.05, no meaningful results were found. Consequently, we decided to forgo group-level corrections and instead employed a more stringent p-value threshold of 0.01. Additionally, correlations between the scores from the SSI, BHS, RFL, BPAQ, BDI, STAI-1, and STAI-2 questionnaires and the activation maps were computed using MATLAB 2021b.

## Results

### Demographic features

The demographic characteristics of the study participants are presented in Table 1. No significant differences were found among the three groups in terms of gender (p = 0.78), age (p = 0.76), marital status (p = 0.43), or education level (p = 0.24).

**Table 1:** Demographic Features of the Study Sample.

Groups Qualitative variables		p-value	SA + MDD Frequency (%) (N = 20)		MDD Frequency (%) (N = 20)		HC Frequency (%) (N = 20)	
Gender	Male Female	0.782	3 (15.0%) 17 (85.0%)		3 (15.0%) 17 (85.0%)		4 (20.0%) 16 (80.0%)	
Marital	Married Unmarried	0.438	12 (60.0%) 8 (40.0%)		13 (65.0%) 7 (35.0%)		10 (50.0%) 10 (50.0%)	
Education	Diploma, under diploma College degree	0.24	13 (65.0%) 7 (35.0%)		8 (40.0%) 12 (60.0%)		9 (45.0%) 11 (55.0%)	
**Quantitate variables**		**p-value**	**Mean (SD)**	**Age range**	**Mean (SD)**	**Age**	**Mean (SD)**	**Age**
Age		0.76	34.10 (10.53)	16-57	35.25 (10.19)	17-57	34.40 (9.35)	20-55

### Clinical analysis

The Kolmogorov–Smirnov test indicated that all clinical variables were normally distributed. ANOVA revealed significant differences in all clinical variables among the three groups. However, post hoc analysis showed that only the SSI and RFL scales differed significantly between the SA + MDD and MDD groups (*p* < 0.05). Although the mean scores for hopelessness, anxiety (both one-month and lifetime), aggression, and depression severity were higher in the SA + MDD group compared to the MDD group, these differences were not statistically significant (*p* > 0.05). Detailed results are presented in Table 2.

**Table 2:** The Dunnett Post Hoc Analysis of Clinical Variables in the Three Study Groups.

*Variable*	*Groups*		*Mean diff.*	*SE*	*p-value*
BHS	SA + MDD	MDD	2.24	1.16	0.140
		HC	10.99	1.16	<0.001
	HC	MDD	−8.75	1.13	<0.001
SSI	SA + MDD	MDD	15.77	2.05	<0.001
		HC	23.37	2.24	<0.001
	HC	MDD	−7.59	2.24	0.004
RFL	SA + MDD	MDD	−69.52	10.97	<0.001
		HC	−101.27	10.97	<0.001
	HC	MDD	31.75	10.67	0.012
BPAQ	SA + MDD	MDD	4.99	6.15	0.698
		HC	15.64	6.15	0.037
	HC	MDD	−10.65	5.99	0.187
STAI-1	SA + MDD	MDD	2.28	3.23	0.761
		HC	24.43	3.23	<0.001
	HC	MDD	−22.15	3.15	<0.001
STAI-2	SA + MDD	MDD	2.93	2.87	0.567
		HC	23.58	2.87	<0.001
	HC	MDD	−20.65	2.80	<0.001
BDI	SA + MDD	MDD	1.58	1.57	0.578
		HC	19.18	1.57	<0.001
	HC	MDD	−17.60	1.53	<0.001

### Behavioral analysis

Table 3 presents the ANOVA results comparing reaction time (RT) and number of correct responses (NCR) across the three study groups. For NCR, the group effect yielded an F-value of 0.94, indicating no statistically significant difference among the groups (*p* = 0.397). The number

**Table 3:** ANOVA Results for Reaction Time (RT) and Number of Correct Response (NCR) across the Three Groups.

	Predictor	Sum of Squares	df	Mean Squares	F-value	p-value
**ACC**	Groups	7.92	2	3.96	0.94	0.397
	Error	180.79	43	4.20		
**RT**	Groups	81.97	2	40.98	3.85	0.02
	Error	457.35	43	10.63		

RT = Reaction Time measured in milliseconds, NCR = Number of Correct Responses, df:Degrees of Freedom

BHS: Beck hopeless scale, SSI: Suicide scale ideation, RFL: Reason for life, BPAQ: for aggression, STAI-1: Spielberger Anxiety State-Trait Inventory for last month, STAI-2: Spielberger Anxiety State-Trait Inventory for lifetime, BDI: Beck depression inventory, diff.: difference, SE: standard error

of correct responses was 5 (22%) for the MDD group, 5 (24%) for the SA + MDD group, and 4 (20%) for the HC group. In contrast, the analysis of RT revealed a significant group effect (F = 3.85, *p* = 0.02). To further examine this difference, post hoc pairwise comparisons were conducted using one-tailed independent *t*-tests (Table 4). No significant differences in RT were found between the MDD and HC groups (*p* = 0.18) or between the SA + MDD and HC groups (*p* = 0.90). However, a significant difference in RT was observed between the SA + MDD and MDD groups (*p* = 0.04).

**Table 4:** Pairwise Comparisons of Reaction Time (in seconds) between the Study Groups.

Comparison	T	df	p-value	Mean MDD	Mean HC
**MDD vs HC**	0.89	31	0.18	14.17	13.31
**SA + MDD vs HC**	−1.72	27	0.04	10.9	13.31
**SA + MDD vs MDD**	−2.70	28	0.005	10.9	14.17

*df:Degrees of Freedom, T:T-Test, P:p-value, SE: standard error*

### Neuroimaging results

A significant decrease in activation was observed in the left medial superior frontal cortex and left anterior cingulate cortex in the SA + MDD group compared to the HC group.

**Fig 4:**
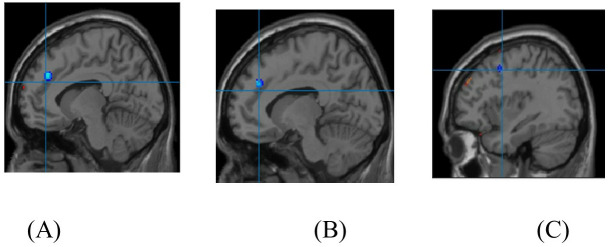
Brain regions with significantly decreased activations for the Raven task are shown in blue. (A) Left Medial Superior Frontal (B) Left Anterior Cingulate and (C) includes Left Precentral.

Furthermore, the SA + MDD group also showed significantly reduced activation in the left medial superior frontal cortex, left anterior cingulate cortex, and left precentral gyrus compared to the MDD group, as shown in Fig 4. Tables 5–7 present the neuroimaging results for the three study groups.

**Table 5:** ANOVA Test Results for Brain Regions Activation during the Raven Task.

Task	Areas	df	Sum squared	Mean squared	Absolute- F scores	p-value
**Raven**	Left Medial Superior Frontal	2	24.966	12.483	8.488	<=0.001
	Left Anterior Cingulate	2	21.631	10.815	7.282	<=0.001
	Left Precentral	2	38.629	19.315	6.045	<=0.001

**Table 6:** Mean and Standard Deviation of T Values in Regions with Significant Differences among the Three Study Groups.

Task	Areas	SA + MDD (Mean ± SD)	MDD (Mean ± SD)	HC (Mean ± SD)
**Raven**	Left Medial Superior Frontal	0.444 ± 1.325	−1.053 ± 1.304	−1.011 ± 1.048
	Left Anterior Cingulate	0.134 ± 1.603	−1.279 ± 1.529	−0.923 ± 1.070
	Left Precentral	0.607 ± 1.529	−1.091 ± 1.435	0.220 ± 1.969

**Table 7:** Areas with Significant Differences Between the Three Study Groups and Their Corresponding Features in the Raven task (SA + MDD vs HC, and SA + MDD vs MDD).

Task	Pairwise group comparison	Areas	MNI coordinates	F
**Raven**	SA + MDD < HC	Left Medial Superior Frontal	(−12, 37, 31)	−8.48
		Left Anterior Cingulate	(−5, 39, 28)	−7.28
	SA + MDD < MDD	Left Medial Superior Frontal	(−12, 37, 31)	−8.48
		Left Anterior Cingulate	(−5, 39, 28)	−7.28
		Left Precentral	(−54, −5, 47)	−6.04

## Discussion

One of our key findings was that the SA + MDD group had significantly lower scores on the SSI and RFL scales compared to the MDD group. Although the SA + MDD group exhibited higher levels of hopelessness, anxiety, and aggression than the MDD group, these differences were not statistically significant. Another significant finding was decreased activity in three brain areas—the ACC, medial SFC, and precentral gyrus—all located in the left hemisphere of participants with SA + MDD. Additionally, there was no significant difference in the number of correct responses among the three groups, suggesting that the task difficulty was comparable, as their IQ levels did not differ significantly. However, it is worth noting that the SA + MDD group showed a significantly shorter reaction time. This may suggest that, due to their severe negative emotional state, individuals in this group might not have fully engaged in the cognitive processing needed to solve the problem effectively. Instead, they may have responded quickly, relying on chance.

### Anterior cingulate cortex

The reduced activity observed in the anterior cingulate cortex (ACC) among individuals with SA + MDD, compared to healthy controls (HC), may be due to the complex interaction between neural circuits involved in cognitive processes and emotional regulation. The ACC plays a crucial role in problem-solving and decision-making, acting as a hub for processing emotional information and regulating cognitive control functions [26,27]. In individuals with a history of recent suicide attempts, this disruption in ACC activity could impair the balance needed for effective problem-solving. Dysregulation in the ACC has been linked to impaired cognitive conflict monitoring, response inhibition, emotional regulation, impulsivity, and insight problem-solving [6,28–31]. According to Anderson et al. [32], the ACC establishes subgoals that enable different paths of information processing when individuals face otherwise identical problem situations. These subgoals determine which path is chosen at decision points in information processing. Additionally, ACC activation increases upon solution retrieval, reflecting the cognitive engagement required to process it [32]. An fMRI study revealed that suicide attempters exhibited decreased activation in the anterior cingulate during cognitively challenging tasks, especially compared to depressed individuals who had not attempted suicide. This was observed specifically during the response inhibition phase of a go-no-go task, which is in line with our results [29]. Therefore, reduced ACC activity in those with a history of suicide attempts could compromise problem-solving, reasoning, and decision-making abilities, potentially decreasing the likelihood of successfully completing Raven’s task.

Furthermore, in our study, the SA + MDD group scored higher on scales measuring hopelessness and anxiety compared to the other groups, although these differences were not statistically significant. This suggests that these individuals may be experiencing a more severe emotional state. Additionally, participants with SA + MDD exhibited hypoactivity in the ACC compared to the other two groups, which is a key center for emotional regulation [33]. Previous studies have shown that a lack of emotion regulation skills is associated with suicidal ideation [34,35]. The risk of a repeat suicide attempt is particularly high in the first year after discharge from the hospital following a suicide attempt [36,37]. Our participants, who had attempted suicide within the last 1–4 weeks, are at elevated risk of subsequent attempts. This is consistent with the observed impulsivity, severe emotional distress, and impaired cognitive control in this group.

### Medial superior frontal cortex

Another finding of our study was a significant decrease in left medial superior frontal cortex (SFC) activity in the SA + MDD group compared to the MDD and HC groups. The medial SFC, which includes the supplementary motor area (SMA) and pre-supplementary motor area (preSMA), plays a critical role in cognitive control [38]. A comprehensive review of 38 fMRI studies indicated that the preSMA is closely associated with decision uncertainty and response errors [39]. In individuals with SA + MDD, reduced activation in the left medial SFC may impair cognitive control mechanisms that regulate suicidal impulses. Poor cognitive control—characterized by increased decision uncertainty and response errors—can negatively affect decision-making and problem-solving abilities. This was reflected in the SA + MDD group’s poorer performance on the Raven’s Progressive Matrices task.

Cognitive models suggest that individuals with negative self-perceptions are more prone to experiencing suicidal thoughts and engaging in suicidal behaviors [40,41]. Moreover, having suicidal thoughts may exacerbate the negative self-perceptions that are commonly seen among individuals with depression [42]. In line with our findings, an EEG study was conducted on adolescents who had experienced suicidal thoughts or had attempted suicide within the past year. The results showed that individuals who had attempted suicide exhibited higher P2 amplitudes in response to negative words compared to positive words, suggesting heightened attention and arousal in response to negative self-referential stimuli [43]. Conversely, an increase in negative thoughts and depressed mood may impair SFC functioning, reducing the ability to generate effective solutions and increasing negative self-referential thinking. Such individuals may become more attuned to negative connotations and self-relevant feedback. Our study demonstrated that the left medial SFC showed reduced functionality during problem-solving tasks, as evidenced by performance on the Raven’s Progressive Matrices.

### Precentral gyrus

The precentral gyrus is involved in executive functions, particularly in planning and response inhibition [44]. It is also connected to other motor regions, such as the pre-supplementary motor area, which plays a crucial role in inhibitory control [45,46]. Difficulties in inhibitory control have been associated with a higher likelihood of acting on suicidal or aggressive thoughts [47]. Therefore, decreased activity in the precentral gyrus in individuals with a history of suicide attempts could impair their problem-solving ability and reduce the likelihood of successfully completing the Raven’s task. Previous research has also suggested that abnormalities in the precentral cortex may increase the risk of suicide attempts in individuals with major depressive disorder (MDD) [48,49].

The dorsolateral prefrontal cortex (DLPFC) is involved in various cognitive functions, including working memory, shifting attention between sources of information, inhibiting task-irrelevant processing, and problem-solving [50,51]. Altered activity in this region has been observed in individuals exhibiting suicidal behavior [52]. In a study by Elliott et al., six individuals with major depression and six healthy controls performed the Tower of London task while undergoing PET imaging. The results showed significantly reduced activation in the dorsolateral prefrontal cortex and caudate nucleus among depressed individuals during low-difficulty tasks. Additionally, depressed participants showed reduced activation in the anterior cingulate cortex (ACC) during high-difficulty tasks [53]. Our study focused exclusively on high-difficulty tasks; consequently, we did not observe changes in DLPFC or caudate nucleus activation. However, we observed decreased ACC activity during these challenging tasks.

## Conclusion

In conclusion, our findings suggest that individuals who have recently attempted suicide exhibit reduced neural activity in specific areas of the left hemisphere while engaging in problem-solving and reasoning tasks, including the ACC, medial SFC, and precentral gyrus. To address this, neuromodulation treatments such as repetitive transcranial magnetic stimulation (rTMS) targeting the left hemisphere may be beneficial for individuals with recent suicide attempts. Furthermore, ketamine, which exerts its effects through NMDA receptor antagonism [54], may help stimulate the reward system in the left hemisphere and potentially reduce the risk of future suicide attempts.

## Limitations

One limitation of our study was the high difficulty level of the Raven task, which may have contributed to the absence of significant differences in behavioral performance among participants. It is possible that using a less demanding task to assess problem-solving could have revealed behavioral differences between the SA + MDD, MDD, and HC groups. This may also have provided greater insight into potential right hemisphere involvement. Another limitation is that participants were recruited from the hospital’s poisoning department. Since the study included individuals who had attempted suicide within the previous 1–4 weeks, acute physiological or psychological factors may have influenced their brain function during neuroimaging.
